# Detection and Genetic Characterization of Relapsing Fever Spirochete *Borrelia miyamotoi* in Estonian Ticks

**DOI:** 10.1371/journal.pone.0051914

**Published:** 2012-12-14

**Authors:** Julia Geller, Lidia Nazarova, Olga Katargina, Lilian Järvekülg, Natalya Fomenko, Irina Golovljova

**Affiliations:** 1 National Institute for Health Development, Tallinn, Estonia; 2 Department of Gene Technology, Faculty of Science, Tallinn University of Technology, Tallinn, Estonia; 3 JSC VECTOR-BEST, Novosibirsk, Russia; 4 Institute of Chemical Biology and Fundamental Medicine SB RAS, Novosibirsk, Russia; Cornell University, United States of America

## Abstract

During the years 2008–2010 *I. ricinus* and *I. persulcatus* ticks were collected from 64 sites in mainland Estonia and on the island Saaremaa. Presence of *B. miyamotoi* was found in 0.9% (23/2622) of ticks. The prevalence in *I. persulcatus* and *I. ricinus* ticks differed significantly, 2.7% (15/561) and 0.4% (8/2061), respectively. The highest prevalence rates were in found South-Eastern Estonia in an area of *I. persulcatus* and *I. ricinus* sympatry and varied from 1.4% (1/73) to 2.8% (5/178). Co-infections with *B. burgdorferi s.l.* group spirochetes and tick-borne encephalitis virus were also revealed. Genetic characterization of partial 16S rRNA, p66 and glpQ genes demonstrated that Estonian sequences belong to two types of *B. miyamotoi* and cluster with sequences from Europe and the European part of Russia, as well as with sequences from Siberia, Asia and Japan, here designated as European and Asian types, respectively. Estonian sequences of the European type were obtained from *I. ricinus* ticks only, whereas the Asian type of *B. miyamotoi* was shown for both tick species in the sympatric regions.

## Introduction

The *Borrelia* genus consists of two groups of species [1].The Lyme borreliosis (LB) group of spirochetes include agents that cause disease (LB) in humans as well as some species not associated with human disease. The LB group organisms are widely spread in Europe and North America and transmitted between vertebrates by hard (ixodid) ticks [2]. The relapsing fever (RF) group spirochetes mainly use soft (argasid) ticks as vectors [3] but some of them are transmitted also by hard tick vectors. This group includes *B. theileri*, which is vectored by *Rhipicephalus* ticks and causes infections in large livestock, *B. lonestari,* which is transmitted by *Ambylomma americanum* and causes infections in deer [4], as well as *B. miyamotoi*, which is transmitted by *Ixodes* ticks and is found in a small percentage of ticks in Eurasia and North America [5], [6], [7], [8], [9].

*B. miyamotoi* was isolated for the first time in Japan in 1995 from *I. persulcatus* ticks as well as from blood of *Apodemus argenteus* mice [7], [10]. DNA of closely related spirochetes was subsequently detected in *I. scapularis* [11] and *I. pacificus* [12] in the USA. In Europe, *B. miyamotoi* was detected in *I.ricinus* ticks in Sweden [6] and Germany [9]. In European and Asian regions of Russia DNA of *B. miyamotoi* was detected in *I.persulcatus* [13] and *I.ricinus* as well as in human blood [8]. In addition to *A. argenteus*, it has been shown that white-footed mice (*Peromyscus leucopus*) may serve as host reservoirs for *B. miyamotoi* [11] and detection of *B. miyamotoi* from wild turkeys (*Meleagris gallopavo*) was also recently reported [14].

Unlike LB spirochetes, *B. miyamotoi* and other relapsing fever spirochetes are vertically transmittable from a female adult tick to her offspring [15], [16], [17]. Also transmission of spirochetes by co-feeding from nymph to larvae and horizontal transmission from infected mice to ticks was experimentally shown [4], [12], although at a lower rate compared to *B. burgdorferi* s.l. [11].

Over the last decade *B. miyamotoi* has been detected in *Ixodes* ticks in the USA [11], [12], Sweden [18], Czech Republic [16], France, and Germany [9] as well as in Russia [13], [19], [20]. Human disease caused by this RF group spirochete has not been well characterized, but recently probable cases of *B. miyamotoi* infection in RF-patients were reported from Russia [8], [21], [22].

Our aim was to investigate the presence and the prevalence of *B. miyamotoi* in different areas of Estonia.

## Materials and Methods

### Ethics Statement

According to Estonian legislation no specific permits were required for the described field studies. None of the locations described in the study were situated on the private land, in the National parks nor protected area. The described field studies did not involve endangered or protected species.

### Collection of Ticks

**Figure 1:**
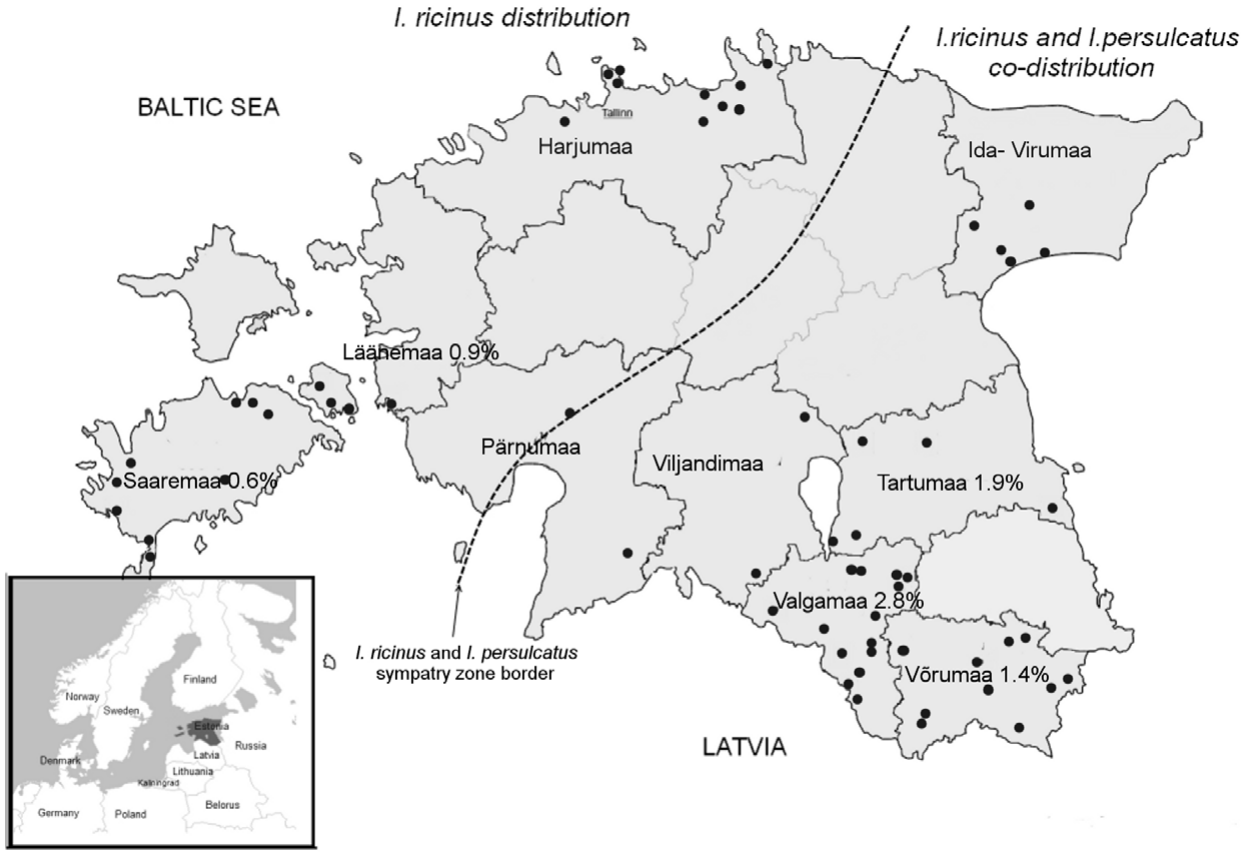
Tick sampling sites in Estonia and the prevalence of *B. miyamotoi*. The areas of *I. ricinus* and *I. persulcatus* distribution and sympatry are indicated. The ticks’ collection sites indicated by dots.

Ticks were collected from the vegetation by flagging from April to November during 2008–2010 at 64 sites in mainland Estonia and on Saaremaa island (Figure 1, Table 1). Tick species were independently identified by morphological criteria by two entomologists, washed in 70% ethanol, rinse twice with sterile PBS and individually stored at −70°C.

**Table 1:** *Borrelia miyamotoi* detection in ticks and estimated prevalence (%).

Place of collection	*I. ricinus*			*I. persulcatus*			Total no. ticks infected/tested (%)	
	No. adultsinfected/tested(%)	No. nymphsinfected/tested(%)	Total no. ticksinfected/tested(%)	No. adultsinfected/tested(%)	No. nymphsinfected/tested(%)	Total no. ticksinfected/tested(%)		
Ida-Virumaa	0/43	0/19	0/62	0/92	0/9	0/101	0/163	*I. persulcatus* and *I. ricinus* sympatric area 17/1324 (1.3%)§
Viljandimaa	0/2	–*	0/2	0/44	–	0/44	0/46	
Tartumaa	0/226	2/66 (3.0%)	2/292 (0.7%)	5/187 (2.7%)	4/94 (4.3%)	9/281 (3.2%)	11/573 (1.9%)	
Valgamaa	0/57	–	0/57	5/121 (4.1%)	–	5/121 (4.1%)	5/178 (2.8%)	
Võrumaa	0/64	–	0/64	1/9 (11.1%)	–	1/9 (11.1%)	1/73 (1.4%)	
Pärnumaa	0/180	0/106	0/286	0/4	0/1	0/5	0/291	
Läänemaa	0/97	1/10 (10%)	1/107 (0.9%)	–	–	–	1/107 (0.9%)	*I. ricinus* area 6/1298 (0.5%)§
Harjumaa	0/217	0/77	0/294	–	–	–	0/294	
Saaremaa	4/508 (0.8%)	1/389 (0.3%)	5/897 (0.6%)	–	–	–	5/897 (0.6%)	
Total	4/1394 (0.3%)	4/667 (0.6%)	8/2061 (0.4%)‡	11/456 (2.4%)	4/104 (3.8%)	15/561 (2.7%)‡	23/2622 (0.9%)	

* Not collected.

‡ P<0.0001 Fisher’s exact and Poisson probability tests.

§ P<0.05 Fisher’s exact test; P<0.001 Poisson probability test.

### Extraction of DNA

Ticks were homogenized in 300 µl of PBS by TissueLyser (Retsch, Haan, Germany). Two hundred microliters of suspensions were used for DNA extraction. DNA was extracted by the guanidinium thiocyanate-phenolchloroform method using the TriPure isolation system (Roche Diagnostics, Lewes, UK) according to the manufacturer’s recommendations. Sterile water was included as a negative control for every DNA preparation set.

### Statistics

Fisher’s exact and Poisson probability tests were used to assess differences in *B. miyamotoi* prevalence in *I. ricinus* and *I. persulcatus* ticks.

### Detection of *B. miyamotoi*, *B. burgdorferi* s.l. and Tick-borne Encephalitis Virus in Ticks

The presence of Borrelia species was detected by amplification of 1256 bp product of 16S rRNA partial gene as described [7] with external primers 16S-Bor-S1F and 16S-Bor-S2R under the following conditions: 35 cycles, 94°C-10 sec, 60°C-1 min, 72°C-90 sec. Nested PCR was performed with primer pair 16S-Bor-S4F and 16S-Bor-S3R [13] and cycling conditions included 35 cycles of initial denaturation at 94°C for10 sec, annealing at 65°C-1 min, elongation at 72°C-90 sec.

To distinguish *B. miyamotoi* from *B. burgdorferi* s.l. among all 16S PCR- positive samples primers targeted *B. miyamotoi* p66 gene were chosen. The amplification of p66 partial gene was performed as described previously [13] with external primers pair M1F and M2R at the following conditions: 35 cycles, 94°C-5 sec, 50°C-10 sec, 72°C-30 sec. A 532 bp product was generated using inner primers M3F and M4R. The annealing time was increased to 15 sec and elongation time to 45 sec.

All p66-positive samples were further used for glpQ partial gene amplification as described by Fomenko et al [13]. Primers Q1F and Q2R were used for the first round of PCR, and cycling conditions included 35 cycles, 94°C–10 sec, 50°C–15 sec, 72°C–35 sec. Inner primers Q3F and Q4R were used in a nested PCR for generation of a 379 bp product at the following cycling conditions: 35 cycles, 94°C–5 sec, 52°C–10 sec, 72°C–30 sec.

To reveal co-infections of ticks with *B. miyamotoi* and *B. burgdorferi* s.l., samples positive for *B. miyamotoi* were amplified by nested PCR for *B. burgdorferi* s.l.-group specific 5S-23S rRNA intergenic spacer (IGS) region as described previously [23], [24] with a modified touch-down program. The first amplification round included 35 cycles, 94°C–1 min, 58°C–1 min and 72°C–2 min, and in the nested PCR, the annealing temperature was decreased to 52°C and amplification was performed for 30 cycles.

Tick-borne encephalitis virus (TBEV) detection was performed by PCR amplification and further sequencing of partial E gene as described earlier [25] with outer primers 283F1 and 827R1 used for the cDNA synthesis and inner primers 349F2 and 814R2 for the second round of PCR amplification.

To confirm the morphological tick species definition, *B. miyamotoi* positive samples were analyzed for mitochondrial 16S rRNA partial gene PCR as described by Caporale et al [26] with further sequencing using primers 16Sa and 16Sb. Cycling conditions included pre-PCR steps 95°C–1 min; 49°C–1 min, 72°C–2 min and 95°C–1 min; 47°C–1 min; 72°C–2 min and amplification 40 cycles, 95°C–30 sec; 45°C–1 min; 72°C–2 min.

PCR products were visualized by electrophoresis in 1% agarose gel stained with ethidium bromide. Deionized water was included in every PCR step as a negative control.

To minimize contamination, reaction mix preparation, sample addition step, amplification and gel electrophoresis were performed in three separate rooms with sterile techniques. Sample addition was performed in a laminar flow cabinet.

### Phylogenetic Analysis

Analysis and alignment of sequences was performed using BioEdit 7.0.0 software. The Maximum Likelihood model was used for phylogenetic tree reconstruction of the partial 16S rRNA (1106 bp), p66 (349 bp and 355 bp for “*I. persulcatus*”-type and “*I. ricinus*”-type, respectively) and glpQ genes (379 bp), using the Tree Puzzle program. 10 000 puzzling steps were applied using the GTP model of substitution for the partial 16S rRNA gene and the Hasegawa-Kishino-Yano (HKY) model for the partial p66 and glpQ genes.

## Results

### Tick Collections and Detection of *B. miyamotoi* DNA

In total, 2622 ticks (1851 adults and 771 nymphs) were collected in 64 sites of 9 Estonian counties, and among them 2061 (78.6%) and 561 (21.4%) were identified as *I. ricinus* and *I. persulcatus*, respectively (Table 1). DNA of *B. miyamotoi* was detected in 23 (0.9%) tick suspensions, 15 of which originated from *I. persulcatus* and 8 from *I. ricinus* ticks. Thus the overall prevalence of *B. miyamotoi* in *I. persulcatus* ticks was 2.7% (15/561) and 0.4% (8/2061) in *I. ricinus* ticks (P<0.0001, Fisher’s exact and Poisson probability tests). The highest prevalence of *B. miyamotoi* in tick populations was detected in the South-Eastern Estonia, in Valgamaa (2.8%), Tartumaa (1.9%) and Võrumaa (1.4%) counties. This region is sympatric for both tick species and DNA of *B. miyamotoi* was found mainly in *I. persulcatus*. However, in other sympatric areas, Ida-Virumaa, Viljandimaa and Pärnumaa, *B. miyamotoi* was not detected. In regions where only *I. ricinus* circulates, the prevalence of *B. miyamotoi* was lower –0.9% and 0.6% in Läänemaa and on Saaremaa island, respectively. Comparison of the prevalence rates of *B. miyamotoi* in areas sympatric for both tick species and areas where only *I. ricinus* circulates demonstrated a statistically significant difference, 1.3% vs 0.5% (P<0.05 Fisher’s exact test and P<0.001 Poisson probability test).

In our study we did not find differences in *B. miyamotoi* prevalence between different tick stages, as *B. miyamotoi* DNA was detected in 1% of nymphal ticks (8 out of 771) and in 0.8% of adult ticks (15 out of 1851).

Co-infection with spirochetes belonging to *Borrelia burgdorferi* s.l. was demonstrated by amplification of 5S-23S IGS, which is specific for the *B. burgdorferi* s.l. group. We showed that 5 ticks (21.7% from all positive ticks) were co-infected with *B. afzelii*, *B. garinii* or *B. valaisiana* (Table 2). Co-infection with another widely distributed tick-borne pathogen, TBEV, was found in adult *I. ricinus* ticks on Saaremaa island. Genetic analysis of the partial E gene sequence revealed that this strain belonged to the European subtype of TBEV.

**Table 2:** *B. miyamotoi* infections in Estonian ticks.

	Place of collection	Species of tick		Type of *B. miyamotoi*	Co-infection with other TBP*
Est1868	Tartumaa	*I. persulcatus*	F	Asian	
Est1885		*I. persulcatus*	F	Asian	
Est3943-4		*I. persulcatus*	F	Asian	
Est1811		*I. persulcatus*	N†	Asian	
Est3486-4		*I. persulcatus*	F	Asian	
Est3487-4		*I. persulcatus*	N	Asian	
Est3698-2		*I. persulcatus*	N	Asian	
Est722-2		*I. persulcatus*	N	Asian	
Est1586		*I. persulcatus*	N	Asian	*B. valaisiana*
Est3115-1		*I. ricinus*	N	Asian	
Est3489-2		*I. ricinus*	F	Asian	
Est4318	Valgamaa	*I. persulcatus*	M	Asian	
Est4350		*I. persulcatus*	F	Asian	
Est4372		*I. persulcatus*	M	Asian	*B. afzelii* (VS461 group)
Est4243		*I. persulcatus*	F	Asian	
Est4412		*I. persulcatus*	F	Asian	*B. garinii* (NT29 group)
Est3633	Võrumaa	*I. persulcatus*	M	Asian	*B. afzelii* (VS461 group)
Est2519	Saaremaa	*I. ricinus*	F	European	
Est3849		*I. ricinus*	M	European	
Est2270		*I. ricinus*	M	European	TBEV-Eu subtype
Est2409		*I. ricinus*	M	European	*B. garinii* (20047 group)
Est2325-3		*I. ricinus*	N	European	
Est1129-4	Läänemaa	*I. ricinus*	N	European	

* Tick-borne pathogen.

† Nymph.

### Genetic and Phylogenetic Analysis of *B. miyamotoi* Sequences

Three genomic regions of *B. miyamotoi*, the partial p66 (532 bp), 16S rRNA (1256 bp) and qlpQ (379 bp) genes, were sequenced for genetic characterization of Estonian samples. Fourteen tick suspensions were amplified for all three genes, 6 for two genes and 3 for one gene region. Analysis of nucleotide sequence similarity of the three genomic regions showed that Estonian samples were divided into two groups: the first with sequences identical to those amplified from *I. ricinus* in Sweden and the European part of Russia (European type) and the second with sequences identical to those found in *I. persulcatus* and human blood in the European part of Russia, Ural and Siberia (Asian type). Within each group, the sequences of the Estonian samples were identical for all three gene regions. Moreover, in the European type cluster, sequences of the partial 16S rRNA, p66 and qlpQ genes amplified in the present study were identical to the *B. miyamotoi* sequences derived from GenBank and detected in Sweden, the European part of Russia, Poland, and France. In the Asian type cluster, the Estonian sequences were identical to those amplified from ticks from different parts of Russia (European part, Ural, Siberia) and also Japan, with the exception of D45192 and AF228023 for the partial 16S rRNA and p66 genes, respectively.

Nucleotide sequence identity between European type and Asian type groups were found 99.4–99.6% for the partial 16S rRNA gene, 98.6% for the partial glpQ gene and 91.6–93.3% for the partial p66 gene. Nucleotide sequences of the partial p66 gene were more diverse and insertion of 6 nucleotides was demonstrated for European type of *B. miyamotoi* when compared to the Asian type.

**Figure 2:**
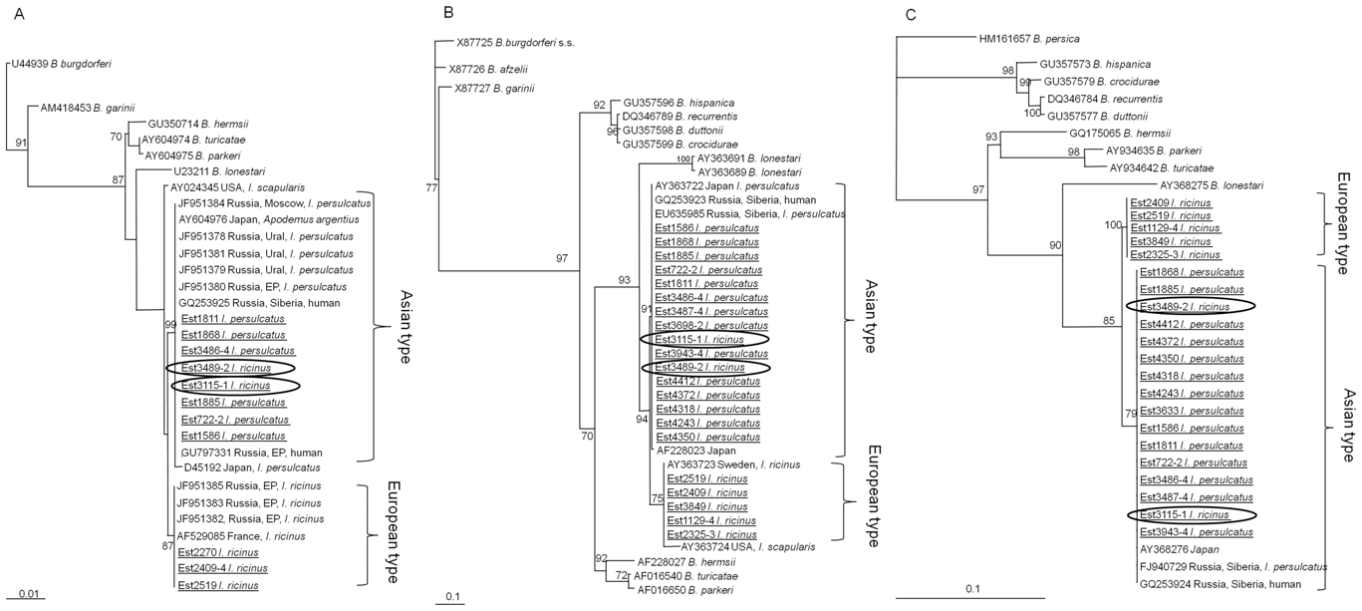
Phylogenetic trees based on the partial sequences of 16S rRNA, p66 and glpQ genes. The Maximum Likelihood model was used for phylogenetic tree reconstruction of the partial A) 16S rRNA (1106 bp), B) p66 (349 bp and 355 bp for “*I. persulcatus*”-type and “*I. ricinus*”-type, respectively) and C) glpQ genes (379 bp). Only quartet puzzling support values >70% are shown. Samples sequenced in the present study are underlined.

On the phylogenetic trees based of the partial p66, partial 16S rRNA and qlpQ genes (Figure 2) sequences detected in the present study clustered with *B. miyamotoi* sequences detected in Siberia and Japan as well as with sequences from Europe and the European part of Russia. Estonian sequences together with previously reported sequences of *B. miyamotoi* formed well supported European type and Asian type clusters. Closely related sequences of *B. miyamotoi* amplified from *I. scapularis* in USA clustered together with the European type of sequences in the phylogenetic tree based on the partial p66 gene sequences, while on tree based on the partial 16S rRNA gene it formed its own lineage albeit with a low bootstrap support.

The two groups of *B. miyamotoi* correspond to the tick species from which sequences were amplified: European type sequences were amplified from *I. ricinus* in Europe and Asian type from *I. persulcatus* in Japan, Siberia and Ural, and additional sequences belonging to this type were detected in blood of patients in Siberia [21] and *A. argenteus* in Japan [7], [10]. In the current study we found the Asian type of *B. miyamotoi* in two *I. ricinus* nymphs (Est3489-2 and Est3115-1) in an area sympatric for both tick species (Tartumaa). Species identification of these nymphs as *I. ricinus* by morphological criteria was confirmed by sequencing of the partial mitochondrial 16S rRNA gene.

## Discussion

In the current study, DNA of relapsing fever spirochetes of *B. miyamotoi* was for the first time detected in ticks in Estonia. We found statistically significant differences between the prevalence rates of *B. miyamotoi* DNA in *I. persulcatus* and *I. ricinus* ticks, 2.7% and 0.4%, respectively. Similar prevalence rates were reported from a sympatric region, Moscow province, at 1.5% in *I. persulcatus* and 0.6% in *I. ricinus* [8]. Previously published data of *B. miyamotoi* DNA detection in *I. persulcatus* demonstrated 2.3–4.5% prevalence in Siberia [13], [19] and 0.9%–16% in Ural [8]. The reported prevalence of *B. miyamotoi* DNA in *I. ricinus* ticks fluctuated from 0.5% in the European part of Russia [8] to 3.5% in Germany [9]. In the USA, similar *B. miyamotoi* prevalence rates in ticks were reported and correspond with those found in Europe: 1.9–2.5% to 6% for *I. scapularis* [11] and 1.7% and 0.7% for *I. pacificus* nymphs and adults, respectively [12]. In all studies where *B. miyamotoi* and *Borrelia* species belonging to LB complex were simultaneously detected, the prevalence rates of *B. miyamotoi* were significantly lower than those of *B. burgdorferi* s.l. [4], [9], [13], [19].

In the current study we found statistically significant differences of *B. miyamotoi* prevalence rates in ticks between the region of *I. ricinus* range (0.5%) and the region sympatric for both tick species (1.3%). This fact may be explained either by a higher tropism of *B. miyamotoi* to *I. persulcatus* ticks or by more favorable conditions for pathogen circulation (microclimate, abundance of small and big mammals and etc.) in sympatric area in Eastern Estonia. However, simultaneous detection of *B. miyamotoi* DNA in both species of ticks collected in the sympatric area, although less frequently in *I. ricinus* compared to *I. persulcatus,* allows us to suggest that the latter explanation is the more probable one. Our investigation of tick-borne encephalitis virus and *Borrelia burgdorferi* s.l. prevalence in ticks demonstrated similar results, with statistically significant differences of prevalence rates between Western and Northern Estonia (areas of *I. ricinus* circulation) and Eastern Estonia (sympatric area for *I. persulcatus* and *I. ricinus*). However, statistically significant differences were not found between the two tick species in the sympatric area (our unpublished data).

In the current study we did not find statistically significant difference in the prevalence of *B. miyamotoi* in adults (0.8%) and nymphs (1%); observations that correspond to findings in the American ticks *I. pacificus* and *I. scapularis* [4], [12] as well as in European *I. ricinus* ticks [9]. Analysis of larval tick stages should be performed for accurate assessment of a cumulative risk of *B. miyamotoi* infection with each subsequent feeding.

In the present study, 21.7% of *B. miyamotoi* positive ticks were also co-infected with spirochetes of the *B. burgdorferi* s.l. genospecies, and in one case a co-infection with TBEV was found. Thus in Estonia *B. miyamotoi* and Lyme disease spirochetes may share hosts, which is in contrast to findings in Germany and France [9] and the USA [4], [11]. Moreover, it has recently been reported that *B. burgdorferi* and *B. miyamotoi* circulate among a separate set of hosts and utilize different transmission loops: for *B. burgdorferi* it is exclusively transmission to susceptible larvae feeding on hosts previously infected by nymphs, while *B. miyamotoi* utilizes mix of vertical and horizontal transmission in the Midwest of the USA [15]. However, *B. miyamotoi* co-infections with *B. garinii* (2.8%) and *B. afzelii* (0.2%) have also been reported in *I. persulcatus* ticks in Siberia [19]. Thus we may suggest that co-feeding on the same host and consequently co-infections of relapsing fever and Lyme disease spirochetes depend on local climatic and environmental conditions and could occur in Estonia and Siberia.

Genetic and phylogenetic analysis of the three gene regions of *B. miyamotoi* revealed that the Estonian sequences divided into two groups, the European and Asian groups, respectively, and that within each group the sequences were identical or shared a high level of similarity in a very large geographical range from Northern Europe (Sweden, Estonia) to the European part of Russia for the first group, and from Estonia, the European part of Russia to Siberia and Japan for the second group. The European type of *B. miyamotoi* sequences have been detected in *I. ricinus* ticks while the Asian has been found in *I. persulcatus* ticks and human blood [5], [6], [7], [8], [13], [27]. In the present study we found that the Asian type of *B. miyamotoi* may be exchanged between tick species in a sympatric area, although not at a very high rate: among 16 sequences of the Asian group, 14 were amplified from *I. persulcatus* and two from *I. ricinus*. Similar results we found for TBEV, sequences belonging to the Siberian subtype of TBEV (TBEV-Sib), which were detected not only in *I. persulcatus* (the natural vector of TBEV-Sib) but also in *I. ricinus* collected in the same sympatric area (our unpublished data).

Recently it has been reported that *B. miyamotoi* probably causes relapsing fever (RF) and Lyme disease-like symptoms in Ural [8] and Siberia [21], and all the reported sequences from patients belonged to the Asian group of *B. miyamotoi*. In Estonia and other parts of Europe human cases of RF caused by *B. miyamotoi* infection have to date not been reported. The reason remains unclear; it may be either underreporting of *B. miyamotoi* infection due to serological cross-reactions in ELISA with *B. burgdorferi* s.l. antigens or a different pathogenecity of the European lineage of *B. miyamotoi*. Further investigations need to be performed in order to understand the vector potential of *I. ricinus* ticks for the Asian lineage of *B. miyamotoi*, which may be useful for the prediction of a possible spread of this group of spirochetes in a westward direction into Europe.
